# A Rare Case of Herpes Zoster with C7 Involvement Mimicking Dermatitis: An Elusive Diagnosis

**DOI:** 10.15190/d.2025.20

**Published:** 2025-12-31

**Authors:** Muhammad Arslan Nizam, Amna Iqbal, Saima Chaudry, Meher Ayyazuddin, Umar Farooq

**Affiliations:** ^1^Department of Medicine, CMH Lahore Medical College, Lahore, Pakistan; ^2^Department of Medicine, Wah Medical College, Wah Cantt, Pakistan; ^3^Department of Medicine, Rawalpindi Medical University, Rawalpindi, Pakistan; ^4^Department of Internal Medicine, Bayonne University Hospital, NJ, USA; ^5^Knights Medical Associates, Bensalem, PA, USA

**Keywords:** Shingles, Herpes Zoster, palmer distribution, atypical presentation, dermatomes.

## Abstract

Herpes Zoster commonly involves thoracic and cranial dermatomal distributions. It is caused by reactivation of the latent varicella-zoster virus (VZV) due to immunosuppression or increasing age. However, uncommon regional presentation for shingles such as the palmar creases, may lead to a misdiagnosis. We report an unusual case of C7 dermatome presentation, prematurely mistaken as contact dermatitis or scabies due to lack of a clustered appearance. Prevention with vaccination is key and so is avoiding delays in diagnosis due to late complications that may arise.

## Introduction

Herpes zoster, commonly known as shingles, occurs due to reactivation of the varicella zoster virus. It typically presents as a band-like distribution of vesicles along a single dermatome, usually on the trunk along the thoracic dermatome. Involvement of the palm is rare, particularly when the brachial plexus is affected^1,2^. Older adults, especially those aged 60-70 years, are more susceptible to viral reactivation due to age-related decline in immune function^3^. This case reports unilateral C7 involvement of herpes zoster which may be misdiagnosed as eczema, irritant contact dermatitis and insect bites such as scabies, due to its atypical presentation of delayed vesiculation^4^. However, the clinical presentation in this patient was compatible with shingles due to the nature of pain and unilateral location following a clear dermatomal pattern. This case presents a rare finding of shingles in the C7 dermatome. Clinicians should perform a thorough physical examination to distinguish shingles-induced pain from other differentials due to risk of misdiagnosis and delayed treatment.

## Case Presentation

A 65-year-old male presented with a 3-day history of fatigue, body aches, anorexia, and burning pain with numbness in his left forearm and hand. An erythematous, pruritic papular rash appeared in the same distribution one day prior. He had no recent trauma, illness, or immunization, and no history of diabetes, immunosuppression, or corticosteroid use. He had a childhood history of varicella but had never experienced shingles and had not received the shingles vaccine. The patient has a stressful job as a diner employee.

On examination, erythematous papules with scattered vesicles were noted on the palmar surface of the left hand - beneath the index and middle fingers - as well as the dorsal hand and forearm. Lesions were scattered rather than clustered, and there were no bullae or significant tenderness. The Neurologic exam, including strength and reflexes, was unremarkable.

Although the distribution was unusual, dermatology consultation confirmed a diagnosis of herpes zoster involving the C7 dermatome - an exceptionally rare presentation, as cervical involvement is uncommon and C7 is among the least frequently affected dermatomes.

**Figure 1:**
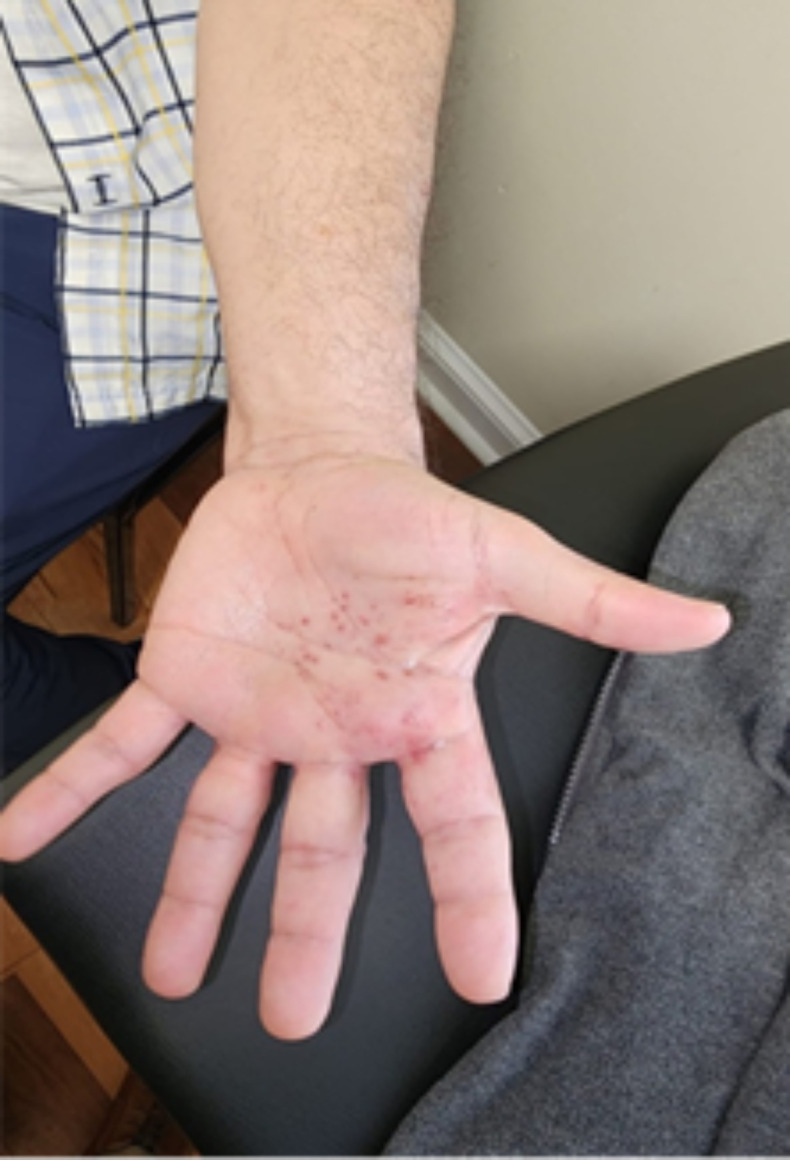
Palmer C7 distribution in Left hand

**Figure 2:**
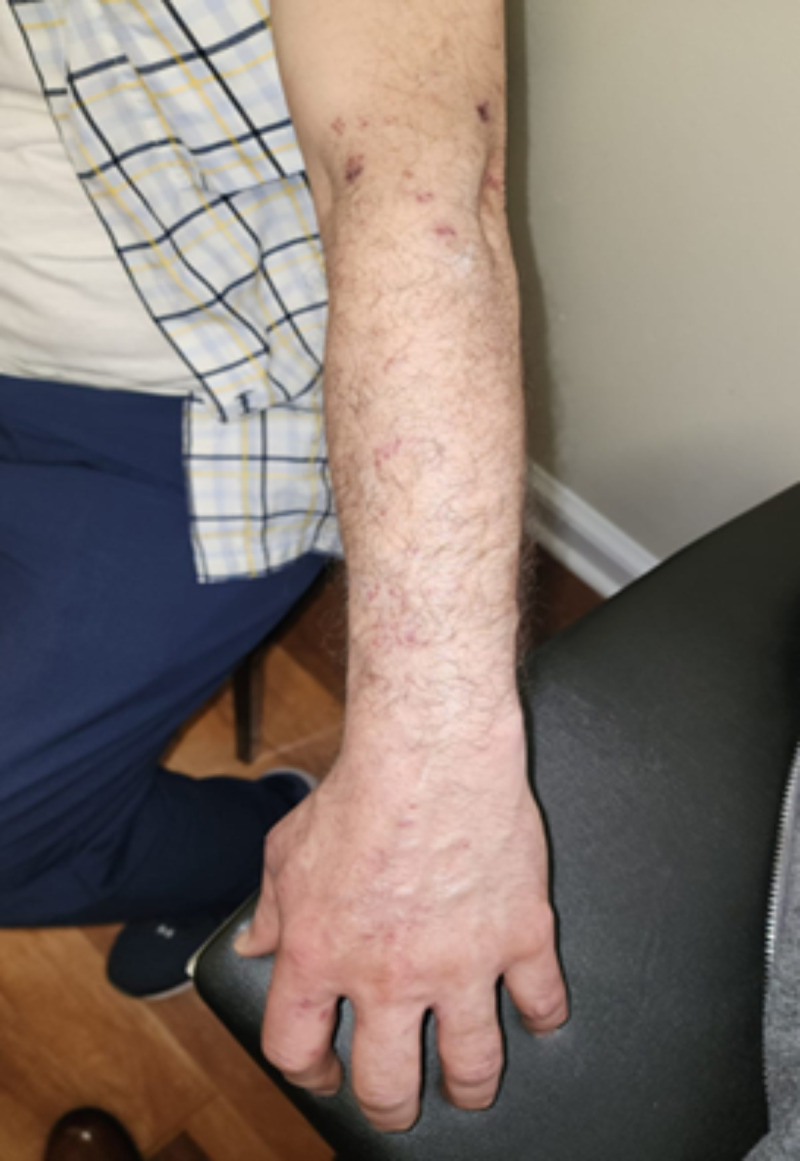
Dorsal aspect of left forearm C7 distribution

**Figure 3:**
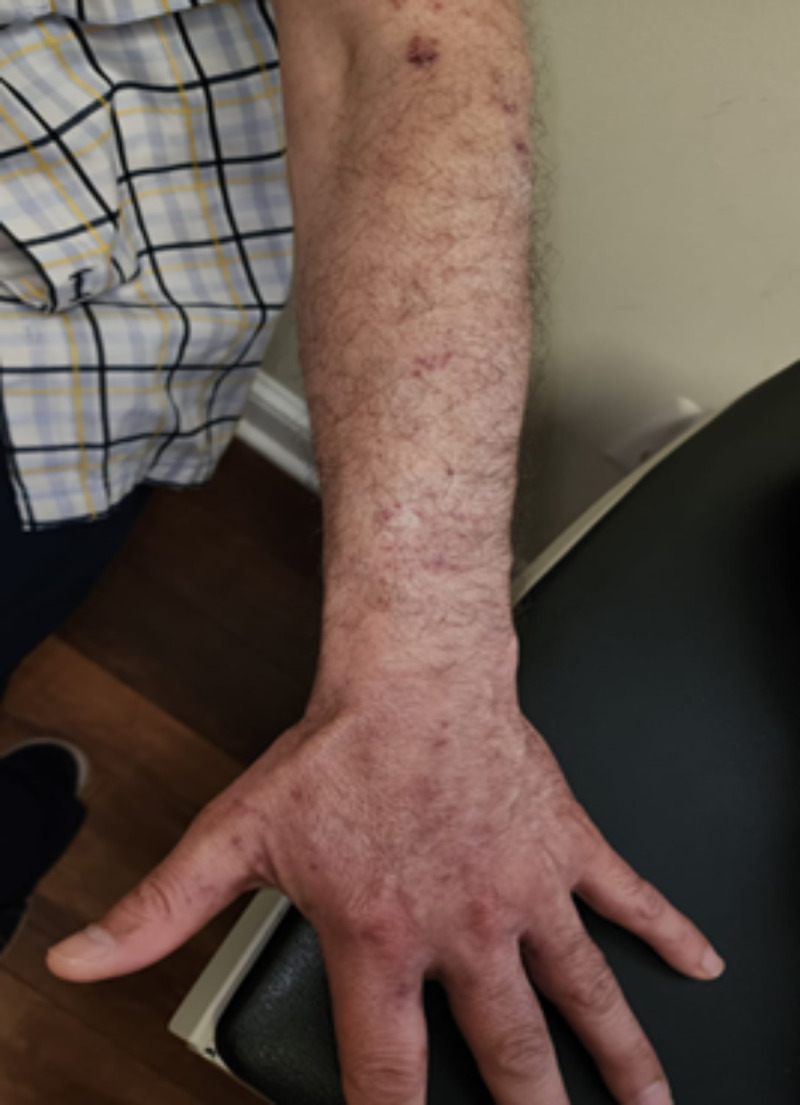
Dorsal aspect of left hand and forearm C7 distribution

The patient was treated with oral acyclovir 800 mg five times daily, gabapentin 100 mg BID, and acetaminophen as needed. At 5-day follow-up, symptoms had significantly improved with crusting of lesions (Figure 1, Figure 2, Figure 3) and no evidence of superinfection or neurologic complications.

## Discussion

The incidence of herpes zoster ranges from 1.2 - 3.4 per 1,000 person-years in younger adults, compared with 3.9 - 11.8 per 1,000 person-years in older adults^4^. Interestingly, epidemiologic data from the United States demonstrate a relatively higher rate of VZV reactivation among younger adults. This trend may be attributable to higher vaccination coverage in older populations, resulting in reduced rates of shingles and postherpetic neuralgia^5^.

This case highlights an atypical presentation of herpes zoster involving the C7 dermatome, initially presenting as a pruritic, erythematous papular eruption following three days of fever, anorexia, and burning neuropathic pain localized to the left forearm and hand. Recognition of the characteristic dermatomal distribution and neuropathic pain ultimately led to the correct diagnosis. Such presentations are commonly misdiagnosed as eczema, irritant contact dermatitis, insect bites, herpes simplex infection, impetigo, candidiasis, or dermatitis herpetiformis^4^.

A comparable presentation was described by Zhang et al., involving a 25-year-old woman who initially developed thermalgia in the left palm, followed by pruritus and burning pain. The patient was initially misdiagnosed with nerve compression syndrome, drug allergy, or poisoning until a careful examination revealed a vesicular eruption on the palmar surface, fingers, and distal index finger, confirming herpes zoster^6^.

Involvement of the ulnar or radial nerve distributions or hand dermatomes is exceptionally rare, with only a limited number of cases reported. Min et al. described shingles involving the palmar cutaneous branch of the ulnar nerve^1^, while Burch et al. reported a middle-aged woman with cubital tunnel involvement and vesicular spread across the ulnar palm and dorsal ulnar forearm^7^. Additionally, Jeevarethinam et al. documented an 83-year-old woman with herpes zoster affecting the C8–T1 dermatome of the right hand^2^.

Psychological stress and excessive workload have been implicated as potential triggers for immune dysregulation and VZV reactivation, particularly in younger patients without traditional risk factors. Cukic et al. described a young patient who developed vesicular lesions along the ulnar nerve distribution in the setting of significant emotional stress and overwork^8^. Similarly, another case reported the onset of herpes zoster in the C5–C6 dermatome approximately 23 days following localized hand trauma^9^. In our patient, who works as a diner employee, occupational stress and physical exertion may plausibly have contributed to viral reactivation.

A review by Chen et al. examining varicella zoster involvement of the shoulder and upper limb found that motor weakness most commonly affects the C5–C7 segments, while L1–L4 involvement predominates in the lower extremities, particularly in cases of segmental zoster paresis^10^. Although neurological complications are uncommon, their possibility underscores the need for vigilant follow-up. Potential sequelae include postherpetic neuralgia, disseminated zoster, VZV vasculopathy, giant cell arteritis, encephalitis, segmental motor weakness, cranial nerve neuropathies, Guillain–Barré syndrome, and multifocal leukoencephalitis^4,5^.

In this case, limited follow-up duration represents a notable limitation and underscores the importance of long-term monitoring to identify delayed neurological or systemic complications.

## Conclusion

We present a patient with an atypical reactivation of varicella zoster involving the C7 dermatome, an uncommon site, particularly in the context of hand involvement. While our report is limited by the absence of further investigations, this aligns with the clinical nature of shingles diagnosis, where additional testing is typically reserved for refractory or complicated cases. This case underscores not only the rarity of zoster affecting the hand but also highlights the limited literature on its occurrence in younger or otherwise healthy individuals. It further raises the possibility of environmental or psychological stressors acting as potential triggers in the absence of traditional risk factors.
